# Cell Wall Biomolecular Composition Plays a Potential Role in the Host Type II Resistance to Fusarium Head Blight in Wheat

**DOI:** 10.3389/fmicb.2016.00910

**Published:** 2016-06-27

**Authors:** Rachid Lahlali, Saroj Kumar, Lipu Wang, Li Forseille, Nicole Sylvain, Malgorzata Korbas, David Muir, George Swerhone, John R. Lawrence, Pierre R. Fobert, Gary Peng, Chithra Karunakaran

**Affiliations:** ^1^Canadian Light SourceSaskatoon, SK, Canada; ^2^National Research Council CanadaSaskatoon, SK, Canada; ^3^Department of Surgery, College of Medicine, University of SaskatchewanSaskatoon, SK, Canada; ^4^Environment CanadaSaskatoon, SK, Canada; ^5^National Research Council CanadaOttawa, ON, Canada; ^6^Agriculture and Agri-Food Canada, Saskatoon Research CentreSaskatoon, SK, Canada

**Keywords:** Fusarium head blight, wheat, type II resistance, Fourier transform infrared spectroscopy, X-ray fluorescence spectroscopy, cell wall, synchrotron

## Abstract

Fusarium head blight (FHB) is a serious disease of wheat worldwide. Cultivar resistance to FHB depends on biochemical factors that confine the pathogen spread in spikes. Breeding for cultivar resistance is considered the most practical way to manage this disease. In this study, different spectroscopy and microscopy techniques were applied to discriminate resistance in wheat genotypes against FHB. Synchrotron-based spectroscopy and imaging techniques, including focal plane array infrared and X-ray fluorescence (XRF) spectroscopy were used to understand changes in biochemical and nutrients in rachis following FHB infection. Sumai3 and Muchmore were used to represent resistant and susceptible cultivars to FHB, respectively, in this study. The histological comparison of rachis showed substantial differences in the cell wall thickness between the cultivars after infection. Synchrotron-based infrared imaging emphasized substantial difference in biochemical composition of rachis samples between the two cultivars prior to visible symptoms; in the resistant Sumai3, infrared bands representing lignin and hemicellulose were stronger and more persistent compared to the susceptible cultivar. These bands may be the candidates of biochemical markers for FHB resistance. Focal plane array infrared imaging (FPA) spectra from the rachis epidermis and vascular bundles revealed a new band (1710 cm^−1^) related to the oxidative stress on the susceptible cultivar only. XRF spectroscopy data revealed differences in nutrients composition between cultivars, and between controls and inoculated samples, with substantial increases observed for Ca, K, Mn, Fe, Zn, and Si in the resistant cultivar. These nutrients are related to cell wall stability, metabolic process, and plant defense mechanisms such as lignification pathway and callose deposition. The combination of cell wall composition and lignification plays a role in the mechanism of type II host resistance to FHB. Biochemical profiling using the synchrotron-based spectroscopy holds potential for screening wheat genotypes for FHB resistance.

## Introduction

Wheat (*Triticum aestivum* L.) is the most extensively grown food crop worldwide (Curtis et al., 2002; Mcmillan et al., 2014) and one of the most important crops in western Canada (Curtis et al., 2002). Fusarium head blight (FHB), caused by the fungus *Fusarium graminearum* [teleomorph: *Gibberella Zeae* (Schwein) Petch] is a devastating disease of wheat due to its negative impact on yield (Walter et al., 2010) and grain quality. The mycotoxin accumulation, such as deoxynivalenol (DON) in cereal grains, results in grain quality issues in food and feed, consequently exacerbating economic losses (Goswami and Kistler, 2004; Osborne and Stein, 2007).

It is generally difficult to control FHB in wheat with any single management tool and an integrated approach using multiple control options is recommended (Krupinsky et al., 2002; Osborne and Stein, 2007). The options used most frequently include genetic resistance and fungicide application. Tillage, crop rotation, and staggered planting of small grain crops may be used to reduce fungal survival on residues (Stack, 2003). Using genetic resistance is the most desirable option; it is practical and can also reduce the need for fungicide application, thus reducing input costs and environmental impact (Von Der Ohe et al., 2010). Current breeding strategies against FHB focus on the combination of desirable agronomic traits and type I and/or type II resistance mechanisms (Bai and Shaner, 2004), which refer to responses against initial infection and spread of the pathogen within the host, respectively (Schroeder and Christensen, 1963). Almost all reports on FHB resistance have been type II, and complete resistance cultivars have not been developed in wheat. The majority of studies have concluded that FHB resistance is quantitative and its inheritance involves several loci on different chromosomes (Buerstmayr et al., 2008; Steiner et al., 2009). Some of these quantitative trait loci (QTL) have been associated with certain transcriptomes and proteomes which may be used as gene markers for plant defense responses against FHB (Bai and Shaner, 2004). However, using these QTL markers for FHB-resistance breeding in wheat can still be difficult due to potentially low yield and quality drag linked to these QTLs (Buerstmayr et al., 2009). The interaction between genotype and environment can further complicate the phenotypic selection, making identification of FHB resistance time consuming and unreliable. Additional selection criteria, complementing genetic markers and phenotypic selection, may improve the efficiency and reliability of FHB resistance screening.

In wheat, symptoms of FHB begin with small water soaked brown spots at either the middle or base of the glume (Goswami and Kistler, 2004; Osborne and Stein, 2007). The discoloration or pre-mature bleaching spreads outwards from the point of infection, and white or magenta mycelium may appear around the edges of infected glumes. Eventually, the majority of inflorescence can become blighted and awns, if present, may become deformed, twisted, and curved downward. Infected kernels or Fusarium damaged kernels (FDKs) are gray and white in color, often with a magenta hue, appear shrunken, and have a floury interior. Many refer these FDKs as “tombstone kernels.” Often the physiological conditions of host plant influenced by nutrition, hydration and plant age can play an important role in FHB development (Osborne and Stein, 2007); wheat crop is highly susceptible between the antithesis and soft dough stage of seed development (Mcmullen et al., 1997; Shaner, 2003). Few studies have characterized biochemical changes in wheat heads, especially in relation to different levels of resistance, during this most susceptible growth stage. Fourier transform mid infrared (FTIR) spectroscopy is a powerful tool for examining biochemical changes during FHB development (Lahlali et al., 2015), which may provide additional criteria for resistance identification. FTIR spectroscopy is a label-free and non-invasive technique that exerts an enormous attraction in biology and medicine, since it offers a rapid way to sample biomolecular contents (Kacurakova et al., 2000; Santos et al., 2010; Largo-Gosens et al., 2014). Its potential for detection and identification of fungal pathogens in plants promises to be of a great value because of the sensitivity, rapidity, low cost, and simplicity (Kummerle et al., 1998; Martin et al., 2005; Erukhimovitch et al., 2010; Peiris et al., 2012; Lahlali et al., 2015). FTIR spectral properties of infected wheat rachis may help reveal compositional differences due to infection between resistant and susceptible wheat cultivars before visible symptoms. The biochemical information may also be related to the type I and II resistance, making the data more versatile. We hypothesize that FHB resistance mechanisms involve type II resistance, which are related to biochemical composition in the cell wall including lignin, pectin, hemicellulose, and nutrients such as calcium, potassium, iron, and zinc in the internodes of wheat rachis.

The objectives of this study were to: (i) identify any structural and anatomical differences in the cell wall of rachis during infection based on microscopy, (ii) assess FTIR spectral absorption between control and inoculated rachis samples of both resistant and susceptible wheat cultivars, (iii) localize the cell compositions on the cross section of rachis of both cultivars with and without fungal infection, and (iv) characterize the nutrients composition in rachis of two contrasting cultivars without and with fungal infection using X-ray fluorescence (XRF) spectroscopy. The goal was to achieve a better understanding of biochemical and nutritional mechanisms related to FHB resistance. Some of the unique spectroscopic bands can be used as biomolecular markers by breeders to identify FHB resistance during routine screening of wheat genotypes.

## Materials and methods

### Fungal culture and inoculum preparation

The wild-type *F. graminearum* (Fg) isolate DAOM 180379 (Canadian collection of fungal cultures, Ottawa, ON) was used in this study. For the production of macroconidia, a plug of actively growing Fg was placed in the center of a petri dish containing Soft Nutrient Agar (SNA). For spore production, cultures were grown in CMC (Carboxymethyl cellulose) medium and incubated at 28°C for 2 days. Conidial suspension was harvested in sterile water, filtered through cheesecloth. A working concentration of 5 × 10^4^ macroconidia/mL was used for inoculation.

### Plant material and inoculation procedures

All experiments were conducted in the environment-controlled growth chamber due to restrictions on using a transformed fungal pathogen in the field. Seeds of resistant “Sumai3” (SU3) and susceptible “Muchmore” (MM) wheat cultivars were sown in peat pots (12.7 cm, diameter) and maintained in a growth chamber at a 20°C: 16°C cycle (day: night), with a 16-h photoperiod until flowering. Pots were watered by hand at the base of the plants. At mid-anthesis, single floret inoculation with the Fg strain was carried out by pipetting 10 μl of the macroconidia suspension (5 × 10^4^/mL) between palea and lemma. Inoculated plants were placed in a dew chamber for 2 days and then moved back to the growth chamber for the rest of the experiment (Lahlali et al., 2015). Plants used as controls were inoculated with a drop of sterile distilled water.

### Visual and microscopic observations of infection

This experiment was designed to compare the anatomy of the rachis nodes, cell arrangement, and cell wall thickness of both resistant and susceptible cultivars as well as the colonization pattern of *F. graminearum*. Cross sections of fresh and frozen rachis nodes were cut using a microtome and mounted on microscope slides for light microscopy. Intact cross sections were chosen to visualize structural and anatomical aspects of the control and inoculated rachis samples using a fluorescence microscope system (Leica Microsystems AF 6000, Leica Canada Ltd. Scarborough, ON). A Zeiss LSM710 confocal microscope (Carl Zeiss Canada Ltd. North York, ON) was used to assess changes in cell wall thickness and epidermis cells at different depths. The tissue samples were optically sectioned at different depth intervals and a Z-stack of images were generated for each fluorescence emission wavelength range. Confocal images were acquired at excitation wavelengths 488, 544, and 633 nm and emissions at 500–550, 573–613, and 650–1000 nm, respectively, for green, red, and blue colors. Images were processed using the Imaris software (Bitplane USA, South Windsor, CT), and histogram stretching and gamma adjustments were used to optimize the visual quality of images.

### Bulk FTIR spectroscopy to characterize the infection and resistance

In order to determine the differences and changes in the biochemical composition of control and infected rachis of both resistant and susceptible cultivars, FTIR spectra of bulk samples were collected at the mid infrared beamline (Canadian Light Source Inc.), using a globar (silicon carbide) as the infrared source. An IFS 66V/S spectrophotometer (Bruker Optics, Ettlingen, Germany) was used with a deuterated triglycine sulfate (DTGS) detector.

Rachis samples from control and inoculated spikes were prepared by the method described before (Naumann et al., 1991). Samples were freeze dried and then ground to fine powder. About 1–2 mg of the powder were homogenized with about 92 mg of dry potassium bromide (KBr) using pestle and mortar, and the mixture was compressed into a pellet. Transmission infrared spectrum was obtained from replicated pellet samples. Each IR spectrum was recorded in the mid infrared range of 4000–800 cm^−1^ wavenumbers at a spectral resolution of 2 cm^−1^. The spectrum of each sample was an average of 64 scans and pure KBr spectra (average of 128 scans) was recorded to normalize all sample spectra. The normalized spectra were then baseline corrected using the rubber band correction (64 points) and vector normalized using the OPUS software (version 7.0, Bruker Optics Inc., Billerica, MA). The FTIR peaks cited in Table 1 were determined using the Quick Peaks routine in OriginPro (version 9.1, OriginLab Corporation, MA) with the settings of local maximum at 0% threshold height, no baseline, and area at *Y* = 0 (Lahlali et al., 2015). The determination of components such as proteins, lignin, cellulose, hemicellulose, and pectin were made by integrating the area under specific bands. The area was determined using the OPUS integration method C, in which the area of interest was determined after considering two baseline points on the left and the other two on the right side of the peak/band. Statistical analysis was performed on integrated areas and ratios of lignin to other bands using ANOVA procedure (SAS Institute, Cary, NC). When the infection effect was revealed to be significant, the LSD test was employed for mean separation at *P* ≤ 0.05.

**Table 1:** **Assignment of bands in the bulk FTIR spectra of rachis of the resistant (Sumai3) and susceptible (Muchmore) wheat cultivars inoculated with ***Fusarium graminearum***^**a**^**.

**Wavenumber (cm^−1^)**	**Biomarker**
1737	Pectin
1655	Proteins (Amide I)
1615–1590	Lignin
1547	Proteins (Amide II)
1515–1505	Lignin
1372	Cellulose
1245	Hemicellulose
1161	Cellulose
1060	Cellulose
930–800	β- glycosidic linkages

a *Dokken et al., 2005; Martin et al., 2005; Mann et al., 2009; Largo-Gosens et al., 2014; Lahlali et al., 2015*.

### Synchrotron-based FTIR spectroscopy and focal plane array imaging

Previous results based on bulk FTIR spectra indicated substantial biochemical differences in the control rachis of resistant and susceptible cultivars (Lahlali et al., 2015). This experiment was designed to better characterize the infection and resistance based on assessing biochemical differences at the cellular level of epidermis using the cross sections of rachis. A synchrotron based FTIR spectroscopy (sFTIR) with a Hyperion 3000 microscope (Bruker Optics Inc., Milton, ON, Canada), equipped with a single element (Mercury Cadmium Telluride, MCT) detector and a 15X objective was used. The spatial resolution of the images was 15 μm × 15 μm. The spectroscopic imaging data were acquired in transmission mode from samples (10 μm thick) mounted on 25 mm diameter and 1 mm thick CaF_2_ slides. Frozen rachis sections were cut with a microtome-cryostat using a diamond knife. The sections deposited on CaF_2_ slides were air dried at room temperature before sFTIR examination. Each sample spectrum was an average of 128 scans and a background of 128 scans was recorded for normalizing all sample spectra. Rachis samples used in this experiment were from 4 days post-inoculation with 10 spectra collected for each sample. Data were analyzed using the principal component analysis as described by Jiang et al. (2015).

In order to assess changes in molecular composition in the large area of rachis cross sections including epidermis, xylem and phloem, the Hyperion imaging system was used (Heraud et al., 2007) with the globar source. The microscope was equipped with a 64 × 64 pixels Focal Plane Array (FPA) detector and a 15X objective. The spatial resolution was 2.7 μm × 2.7 μm per pixel. One FPA grid measurement would result in 4096 spectra (scan area of 184 μm × 184 μm), with each spectrum being an average of 256 scans. Principal component analysis (PCA) was carried out using the Matlab-based program Kinetics (version R2008, Mathworks Inc.), with respective loading graphs generated for epidermis and vascular bundles (Lahlali et al., 2014). Rachis samples were evaluated at 10 days post-inoculation and two replicates were used for each measurement.

### Bulk XRF spectroscopy for nutrients composition in rachis

Until now, there is no report on the involvement of nutrients in the type II resistance to FHB in wheat. The hard X-ray fluorescence data were collected at the Industry Development Education Applications Students (IDEAS) beamline of the Canadian Light Source. Dried rachis samples were mounted inside a vacuum sample chamber equipped with a linear paddle drive. The in-vacuum setup (at about 6 × 10^−1^ Torr pressure) was used to detect low energy fluorescence photons such as phosphorus and sulfur, and to suppress the argon signal from the air that overlaps with potassium peak. Both sides of a rachis segment with 2 nodes long (~10 mm) were exposed to 13 Kev X-rays, and the Acquaman software was used to collect data, with a dwell time of 180 s. The XRF data was normalized initially to the standard ring current of 250 mA, and the XRF spectrum was plotted using the OriginPro software (OriginLab Corporation Inc., USA). Each side of rachis sample was scanned three times at three different regions (six spectra/sample). Background scan of the vacuum chamber with no sample was also recorded. The data were collected from samples collected at 4 and 10 days post-inoculation with two replicates per treatment and a silicon drift detector (Ketek AXAS-M M5T1T0-H0-ML5BEV) was used.

## Results

### Visual and microscopic comparison of infected wheat rachis

**Figure 1:**
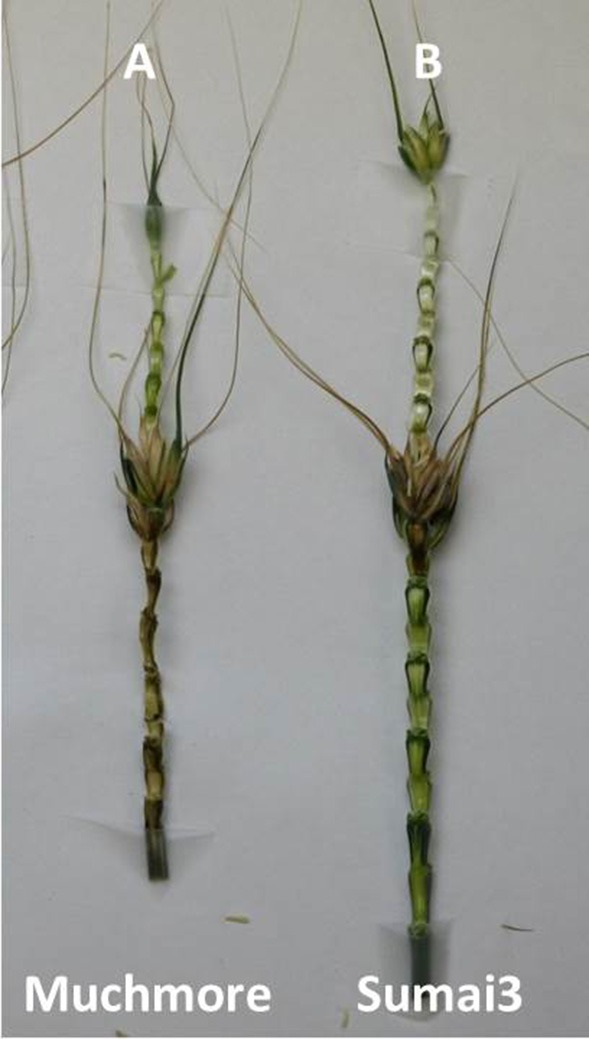
**Symptoms of Fusarium head blight on the rachis of the susceptible and resistant wheat cultivars, Muchmore (A) and Sumai3 (B) at 15 days post-inoculation**.

**Figure 2:**
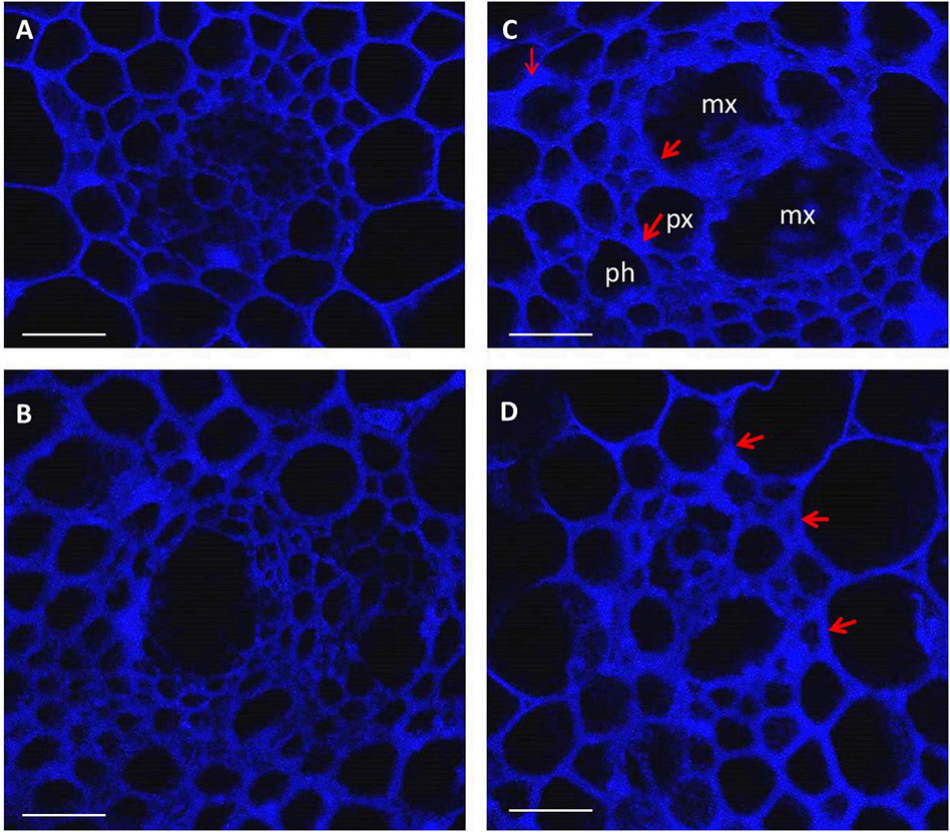
**Images of confocal microscopy on cross sections (10 μm thick) of control (A,B) and inoculated (C,D) rachis of the wheat cultivars Muchmore (A,C) and Sumai3 (B,D) at 4 days post-inoculation with ***Fusarium graminearum*****. The images were created using the emission wavelength of 650–1000 nm (far red range) and the excitation at 633 nm. mx: metaxylem, px: protoxylem, and ph: phloem. Red arrows show changes in cell wall thickness following infection. Scale bar = 50 μm.

Considerable visual differences were observed between infected rachis of resistant and susceptible cultivars at 10 (Figure S1) and 15 days (Figure 1) post-inoculation; those of MM were completely infected whereas those of SU3 were only slightly discolored with browning on the edge (Figure 1, Figure S1). The cross sections of infected nodes under confocal microscopy (Figure 2) showed that the cell wall surrounding meta xylem (mx), protoxylem (px), and phloem (ph) degraded more prominently in MM than in SU3. The cell integrity of SU3 also remained almost intact (Figure 2D). The cell wall was thicker in MM compared to SU3 (Figures 2B,D and Figure S2). No fungal penetration into phloem, xylem, pith or any other cells was observed in infected wheat cultivars, SU3, and MM (Figure 2C and Figure S3).

### Bulk FTIR spectroscopy for potential resistance related biochemical marker identification in rachis

Bands associated with different chemical groups in the fingerprint region (1800–800 cm^−1^), including pectin (C=O at 1740 cm^−1^) and hemicellulose (1248 cm^−1^) were identified and assigned in Table 1. The band at 1655 (Amide I) correspond to C=O and N-H vibrations and at 1546 cm^−1^ (Amide II) to N-H and C-N vibration. The C=C stretching of the aromatic ring of lignin was at 1610-1590 cm^−1^ and 1515-1505 cm^−1^. The cellulose bands were at 1372, 1161, and 1060 cm^−1^, while the band at 995 cm^−1^ was associated with C-C ring vibration.

Based on the absorption in the bulk FTIR spectra, the infection by *F*. *graminearum* caused biochemical changes in the rachis of MM than in those of SU3 (Table 2) relative to the respective controls. In the resistant SU3, the content of the integrated area of the absorption bands arising from C=C stretching of the aromatic ring vibration of lignin (1615–1590, 1460, and 1425 cm^−1^), and hemicellulose (1248 cm^−1^), were persistent after infection. The changes resulted from infection were observed at wavenumbers 1740, 1654, and 1054 cm^−1^, which may be from pectin, proteins, and cellulose, respectively. In the susceptible cultivar MM, however, a decrease in the integrated area of lignin bands, hemicellulose, and cellulose were observed after infection while no change was seen for pectin at 4 days post-inoculation. Meanwhile, an increase was detected for proteins at both 4 and 10 post-inoculations and this may arise from the fungus. The ratio of associated integrated area of lignin (1515 cm^−1^) to pectin (1740 cm^−1^), proteins (amide I), lignin, hemicellulose, and cellulose between inoculated and control rachis were relatively consistent for SU3, but not so much with MM (Table 3). Between inoculated SU3 and MM rachis, the differences in these ratios were more pronounced at 4 than at 10 DPI. The use of the absorption ratios help minimize the variability of data caused by changes in sample thickness in the pellets.

**Table 2:** **Integrated absorption bands in the bulk FTIR spectra of rachis of the susceptible (MM) and resistant (SU) wheat cultivars with and without ***Fusarium gramineraum*** (FHB) inoculation at 4 and 10 days post-inoculation (***n*** = 3)**.

**Absorption bands (cm^−1^)**							
	**Pectin**	**Proteins (Amide I)**	**Lignin**			**Hemicellulose**	**Cellulose**
	**1760–1720**	**1710–1620**	**1615–1590**	**1480–1455**	**1445–1410**	**1261–1200**	**1090–1022**
**MUCHMORE**							
C-4d	7.82^b^ ± 0.14	8.92^a^ ± 0.15	1.31^b^ ± 0.10	2.55^de^ ± 0.2	1.23^d^ ± 0.06	13.67^c^ ± 0.67	107.93^g^ ± 5.37
C-10d	7.97^b^ ± 0.13	10.95^c^ ± 0.19	1.32^b^ ± 0.09	1.98^b^ ± 0.12	0.73^b^ ± 0.05	11.69^b^ ± 1.12	86.50^cd^ ± 2.88
F-4d	7.91^b^ ± 0.20	13.15^e^ ± 0.15	1.27^b^ ± 0.10	2.34^cd^ ± 0.07	1.02^c^ ± 0.06	11.20^b^ ± 0.47	82.50^bc^ ± 3.26
F-10d	6.50^a^ ± 0.09	15.18^f^ ± 0.17	0.80^a^ ± 0.03	1.46^a^ ± 0.06	0.53^a^ ± 0.01	10.16^a^ ± 0.26	70.33^a^ ± 2.62
**SUMAI3**							
C-4d	8.78^d^ ± 0.03	11.03^c^ ± 0.13	**1.26^b^ ± 0.07**	**2.64^e^ ± 0.16**	**1.28^d^^e^ ± 0.07**	**14.40^c^ ± 0.26**	99.92^f^ ± 3.55
C-10d	8.64^d^ ± 0.00	10.95^c^ ± 0.17	**1.29^b^ ± 0.02**	**2.31^c^ ± 0.10**	**0.85^b^ ± 0.04**	**12.05^b^ ± 0.17**	90.90^de^ ± 2.48
F-4d	8.18^c^ ± 0.08	10.61^b^ ± 0.03	**1.25^b^ ± 0.06**	**2.72^e^ ± 0.11**	**1.38^e^ ± 0.15**	**13.53^c^ ± 0.41**	92.18^e^ ± 1.28
F-10^d^	8.26^c^ ± 0.08	11.64^d^ ± 0.25	**1.18^b^ ± 0.1**	**2.37^c^^d^ ± 0.08**	**0.84^b^ ± 0.06**	**11.30^b^ ± 0.23**	79.53^b^ ± 1.64

*C, control not inoculated with FHB; F, Inoculated with FHB; 1740 (1760–1720 cm^−1^); 1654 (1710–1620 cm^−1^); aromatic ring of lignin [1605 (1615–1590 cm^−1^), 1460 (1480–1455 cm^−1^), and 1425 (1445–1410 cm^−1^)]; 1248 (1261–1200 cm^−1^); and 1056 (1090-1022 cm^−1^). Bold indicates most persistent bands in the resistant cultivar after inoculation with FHB. Means in the same column followed by the same letter are not significantly different according to the LSD test P ≤ 0.05*.

**Table 3:** **Ratios between the integrated band of lignin (at 1515 cm^**−1**^) to pectin (1760–1720 cm^**−1**^), proteins (1710–1620 cm^**−1**^), lignin (1615–1590 cm^**−1**^, 1480–1455 cm^**−1**^, and 1445–1410 cm^**−1**^), hemicellulose (1261–1200 cm^**−1**^), and cellulose (1090–1022 cm^**−1**^) in bulk FTIR spectra of the rachis of Muchmore and Sumai3 with and without ***Fusarium graminearum*** infection**.

	**4 DPI**				**10 DPI**			
**Ratios**	**Muchmore**		**Sumai3**		**Muchmore**		**Sumai3**	
	**Control**	**Inoculated**	**Control**	**Inoculated**	**Control**	**Inoculated**	**Control**	**Inoculated**
I1	0.46^d^ ± 0.00	0.37^abc^ ± 0.00	0.48^e^ ± 0.01	0.47^de^ ± 0.02	0.38^c^ ± 0.02	0.34^a^ ± 0.01	0.36^ab^ ± 0.00	0.37^abc^ ± 0.02
I2	0.40^e^ ± 0.00	0.27^c^ ± 0.00	0.38^d^ ± 0.00	0.37^d^ ± 0.02	0.23^b^ ± 0.01	0.15^a^ ± 0.01	0.27^c^ ± 0.00	0.27^c^ ± 0.02
I3	2.74^cd^ ± 0.13	2.21^a^ ± 0.10	3.36^e^ ± 0.11	3.06^de^ ± 0.11	2.40^ab^ ± 0.28	2.77^c^ ± 0.02	2.34^a^ ± 0.06	2.65^bc^ ± 0.33
I4	1.41^c^ ± 0.06	1.25^a^ ± 0.01	1.61^e^ ± 0.06	1.45^c^ ± 0.02	1.54^d^ ± 0.04	1.53^d^ ± 0.01	1.26^ab^ ± 0.02	1.31^b^ ± 0.00
I5	2.90^a^ ± 0.05	2.85^a^ ± 0.07	3.31^b^ ± 0.13	2.9^a^ ± 0.40	4.17^d^ ± 0.17	4.17^d^ ± 0.04	3.43^bc^ ± 0.05	3.70^c^ ± 0.12
I6	0.26^c^ ± 0.00	0.26^c^ ± 0.00	0.29^e^ ± 0.00	0.29^e^ ± 0.01	0.26^c^ ± 0.01	0.22^a^ ± 0.00	0.24^b^ ± 0.00	0.27^d^ ± 0.00
I7	0.03^a^ ± 0.0	0.035^b^ ± 0.00	0.042^c^ ± 0.00	0.041^c^ ± 0.00	0.03^a^ ± 0.00	0.03^a^ ± 0.00	0.03^a^ ± 0.00	0.04^c^ ± 0.00

*Data were collected at 4 and 10 days post-inoculation (n = 3). I1 = 1515/(1760–1720); I2 = 1515/(1710–1620); I3 = 1515/(1615–1590); I4 = 1515/(1480–1455); I5 = 1515/(1445–1410); I6 = 1515/(1261–1200); and I7 = 1515/(1090–1022). Means in the same row followed by the same letter are not significantly different according to the LSD test P ≤ 0.05*.

### Synchrotron-based FTIR spectroscopy and focal plane array imaging for resistance related biochemical marker identification

**Figure 3:**
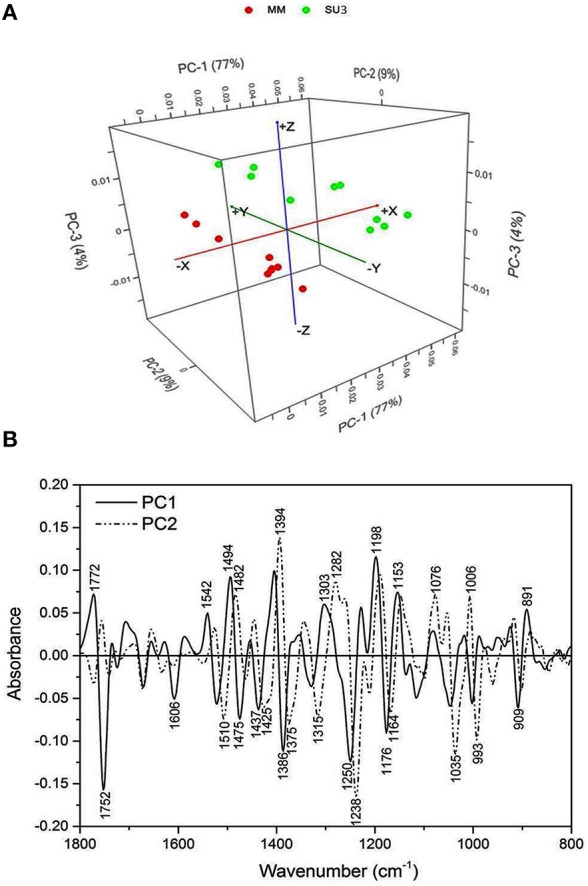
**Principal component analysis of Fourier Transform infrared spectroscopy (sFTIR) of the control rachis epidermis of SU3 and MM**. **(A)**: Score plot and **(B)**: Loading plot for both PC1 and PC2. Rachis samples were sectioned from control samples at 4 days post-inoculation.

The synchrotron-based infrared data from the epidermis of control rachis showed that most of the variations between the cultivars was explained by PC1 and PC2 (86%, Figure 3A). The prominent peaks that contributed to the variations were those encoded for lignin (1606 and 1510 cm^−1^), pectin (1752 cm^−1^), hemicellulose (1250, and 1238 cm^−1^), and cellulose (1394 and 1035 cm^−1^; Figure 3B).

**Figure 4:**
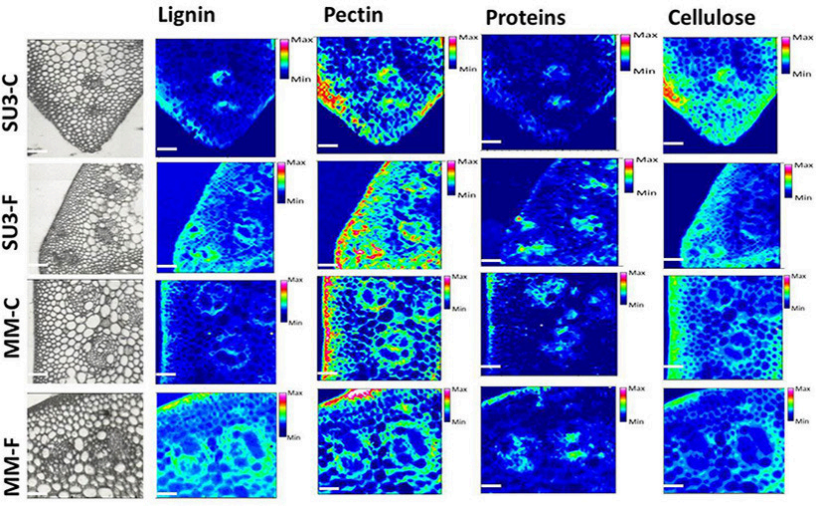
**Optical (left) and infrared images representing the distribution of lignin (1520–1495 cm^**−1**^, 1610–1580 cm^**−1**^), pectin (1745–1715 cm^**−1**^), proteins (1700–1615 cm^**−1**^), and Cellulose (1180–1140 cm^**−1**^) from control and inoculated rachis of resistant (SU3) and susceptible (MM) wheat cultivars with FHB**. Infrared image code: Red-high, blue-low amount of the corresponding imaged component. Scale bar = 100 μm.

**Figure 5:**
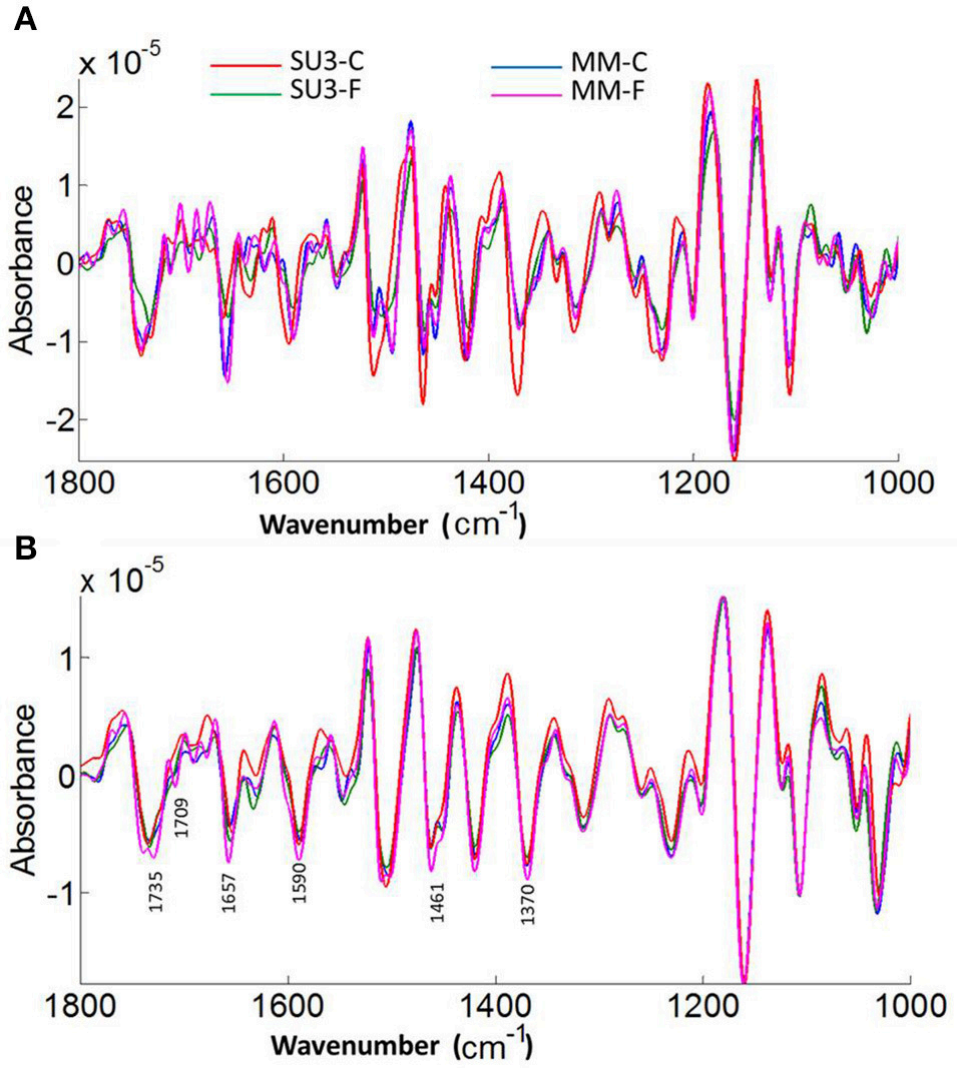
**Averaged second derivatives spectra of control and inoculated epidermis (A) and vascular bundles (B) of rachis from the resistant (SU3) and susceptible (MM) cultivars at 10 days post-inoculation**. The spectra were extracted from the focal plane array (FPA) infrared imaging datasets of rachis cross sections. C, control; F, inoculated with FHB.

**Figure 6:**
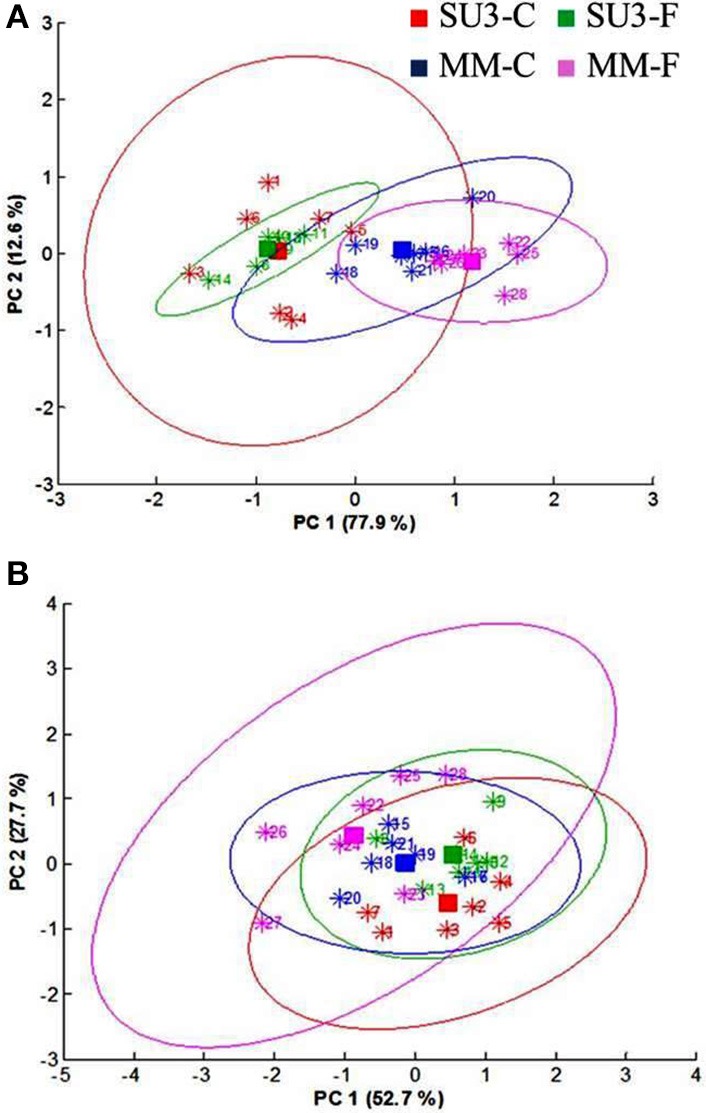
**Principal component analysis (PCA) scores plot of epidermis (A) and vascular bundles (B) of the resistant (SU3) and susceptible (MM) cultivars with (SU3-F, MM-F), and without (SU3-C, MM-C) ***F. graminearum*** inoculation; data from FPA infrared images of rachis cross sections at 10 days post-inoculation**.

**Figure 7:**
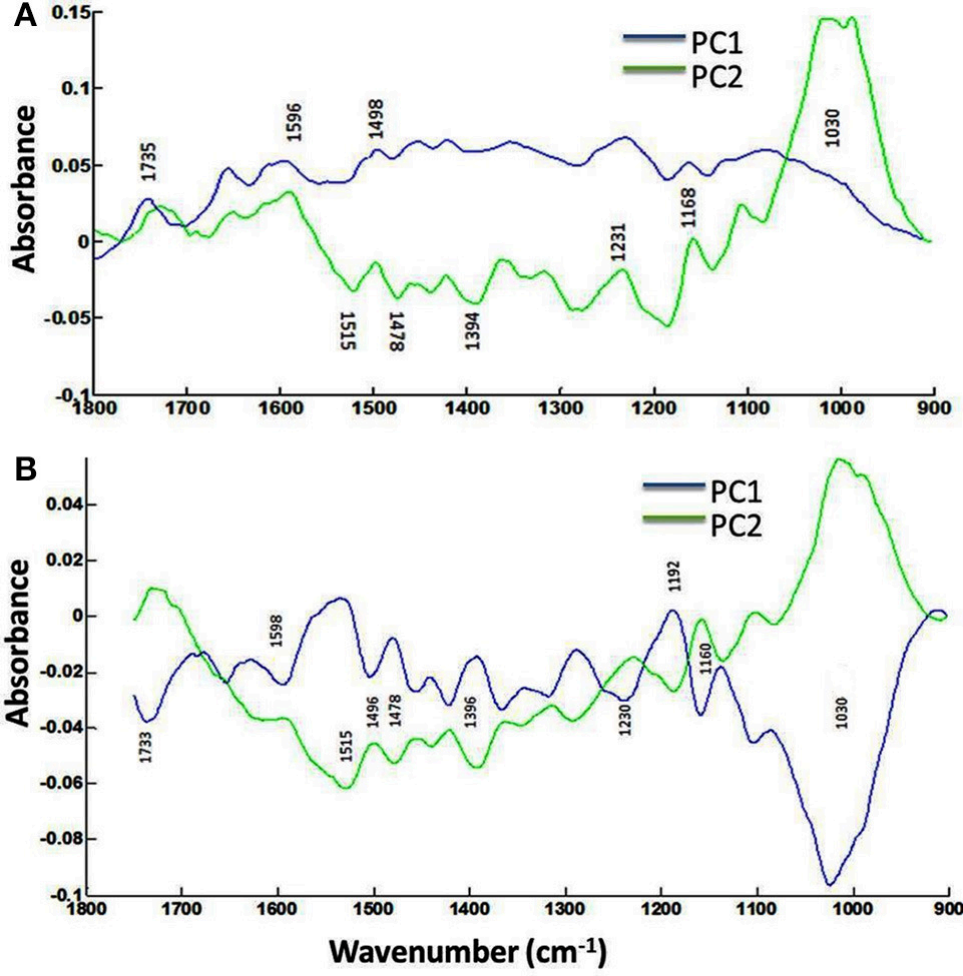
**Loadings plots (PC1 and PC2) of the epidermis (A) and vascular bundles (B) of rachis cross sections of the resistant (SU3) and susceptible (MM) cultivars with the FTIR microspectroscopy**. Data from FPA infrared imaging.

The focal plane array imaging was used to compare epidermis between SU3 and MM (Figure 4), and the infrared images of cross sections from control and inoculated samples confirmed the results of bulk FTIR and sFTIR described above. An increase in lignin and proteins content was observed in epidermal and vascular bundle cells following infection with FHB, while pectin and cellulose decreased. The second derivative spectra in the fingerprint region (1800–800 cm^−1^) showed differences in the epidermal cell wall composition (Figure 5A) and vascular bundles (Figure 5B) between inoculated cultivars. The impact of infection was more significant on the epidermis of MM than that in SU3, where most of the changes were observed at 1735 cm^−1^ (pectin), 1657 cm^−1^ (proteins), 1461 cm^−1^ (lignin), and 1370 and 1160 cm^−1^ (cellulose). However, little change was recorded in vascular bundles following infection with FHB. In the epidermis of MM the band of carbonyl ester (1735 cm^−1^), which is mainly associated to pectin, was converted to the lipid aldehyde (1709 cm^−1^) due to the oxidative stress referred as lipid peroxidation induced potentially by plant defense mechanisms in response to FHB (Shoaib et al., 2013). The score plot for this dataset as a function of two principal components, PC1 (accounting for 78% of variation) vs. PC2 (13%) is shown in Figure 6A. Two distinct clusters are observed; on the positive side of PC1 is susceptible cultivar MM, while on the negative side of PC1 and positive side along PC2 is the resistant cultivar SU3. The loadings plot for PC1 indicates the key differences in wavenumbers that are responsible for grouping the samples along PC1, more specifically the chemical feature that distinguishes the epidermal layer of SU3 rachis from that of MM (Figure 6A). The variables (wavenumbers) with high peak intensities (positive or negative) contributed the most to the separation between the cultivars are associated with pectin (1735 cm^−1^), lignin (1515, and 1478 cm^−1^), cellulose (1498 and 1168 cm^−1^), and hemicellulose (1230 cm^−1^; Figure 7A). In contrast, datasets from vascular bundles did not show substantial differences between the cultivars (Figure 6B); PC1and PC2 together explained 80% of observed variance between infected and non-infected vascular bundles and most of the differences were shown at the wavenumbers associated with pectin, lignin, cellulose and hemicellulose (Figure 7B). The predominance of the bands in aromatic ring of lignin implies cell wall lignification relating to the type II resistance to FHB.

### Bulk XRF spectroscopy for nutrients composition in rachis

**Figure 8:**
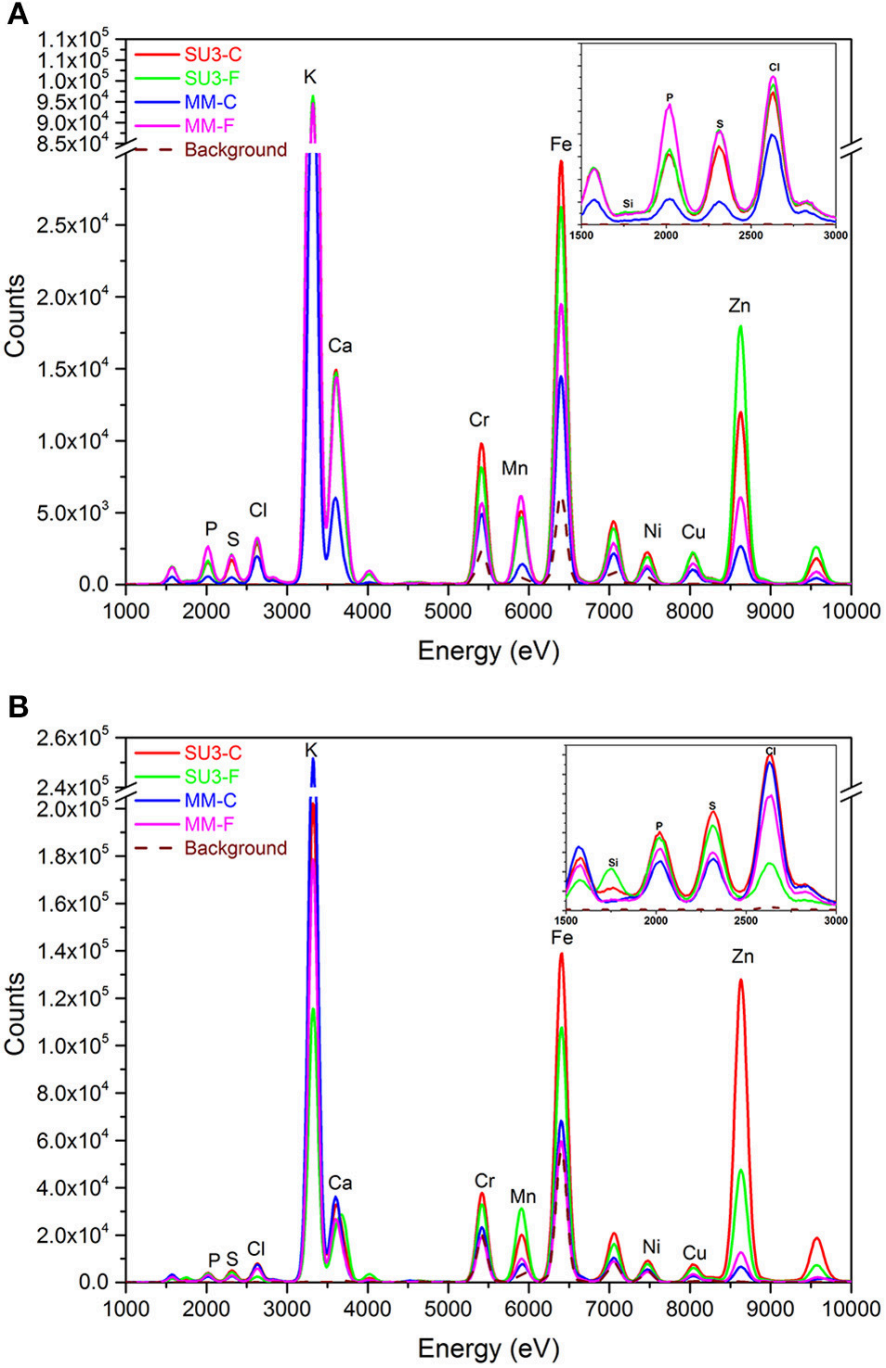
**The XRF spectra of control and inoculated rachis of the resistant (SU3) and susceptible (MM) wheat cultivars at 4 (A) and 10 (B) days post-inoculation, respectively**. Each spectrum represents the average of six datasets.

The nutrients composition in rachis of resistant and susceptible wheat cultivars with and without *F. graminearum* was compared at 4 and 10 DPI (Figure 8) and the average X-ray spectra showed an entire elemental map, with the content of calcium (Ca), iron (Fe), potassium (K), manganese (Mn), and zinc (Zn) being detected readily. In both cultivars, K was present in the highest amount in rachis of both control and inoculated samples, followed by Fe, Zn, Ca, and Mn. At 4 DPI, the rachis of the susceptible cultivar, MM showed lower amounts of Ca, Fe, K, Mn, and Zn, relative to those of SU3 (Figure 8A). Fe concentration decreased while Zn concentration increased in SU3 due to the fungus infection. Both Fe and Zn increased in MM due to the fungus infection. Fe, Zn, and Mn however were present in substantially higher amounts in the resistant cultivar SU3 at 10 DPI than in MM (Figure 8B). Ca was higher in the control SU3 but its concentration changed little with inoculation, while the amount increased substantially in MM at 4 DPI. Small differences were observed for other elements, including phosphorous (P), sulfur (S), chlorine (Cl), and copper (Cu) between the cultivars regardless of inoculation. Interestingly, the silicon (Si) was present only in the resistant cultivar SU3 at 10 DPI and increased after infection with FHB.

## Discussion

Of the five types of resistance to FHB reported in wheat, only three have been extensively studied (Schroeder and Christensen, 1963; Miller et al., 1985), including the type I (to initial infection of spikelets), type II (to spread of infection in spike), and type III (to DON). The modes of action, however, are not well-understood for any of the resistance mechanisms. Because cereal breeders are most interested in the type II resistance against FHB, our study focused on this particular aspect of mechanism using a range of synchrotron-based technologies, including bulk FTIR spectroscopy, sFTIR imaging, focal plane array FTIR imaging, and X-ray spectroscopy to compare the structural, biochemical, and nutritional changes in the rachis internodes. The information will help to identify key factors associated with the resistance and establish additional criteria for efficient screening of wheat germplasms against FHB.

### Visual and microscopic assessment of rachis infected by *F. graminearum*

The infection of rachis leads to destruction of cell wall and starch granules, affecting endosperm storage proteins and consequently resulting in a poor grain quality (Snijders, 2004). Microscopic observations in the current study showed cell wall thickening and cell deterioration surrounding vascular bundles in the inoculated rachis of the susceptible cultivar MM, whereas these changes were not observed for the resistance cultivar SU3. This incompatible interaction in SU3 could be due to the recognition of fungal infection and the defense mechanism can provide a barrier to the pathogen invasion. Several modes of action are possible for the incompatibility. The reinforcement of cell wall can be activated rapidly in response to pathogen penetration and may involve lignituber, callose, silicon, and lignin deposition between the cell wall and membrane directly below the point of penetration (Jacobs et al., 2003; Luna et al., 2011; Bellincampi et al., 2014). In a recent study, a particular composition of lignin-structural component was suggested to play a role in the cell wall reinforcement for SU3 (Lionetti et al., 2015), and the microscopic observations in the current study provide cell and cell wall structure evidence supporting the reinforcement theory. Lignification is a common mechanism for disease resistance in plants (Vance et al., 1980; Bhuiyan et al., 2009). During defense responses, lignin or lignin-like phenolic compound accumulation has been shown in a variety of resistant plant-microbe interactions. Lignification also enables the plant cell wall more resistant to mechanical pressure exerted by penetrating fungal pathogens. Additionally, it can increase the resistance to water, thus lessening the effect of cell wall degrading enzymes from the pathogen (Vance et al., 1980; Nicholson and Hammerschmidt, 1992; Bhuiyan et al., 2009).

### Bulk/synchrotron-based FTIR spectroscopy and focal plane array imaging

The type II resistance to FHB, which is responsible for halting the infection within the spike, is favored by breeders because it can be readily identified under greenhouse conditions (Buerstmayr et al., 2002, 2003). However, it is not well-understood what comprises the type II resistance against FHB. Phenols and triticenes from wheat can be toxic to *F. graminearum* (Spendley and Ride, 1984), although phytoalexines often are discounted for FHB resistance in wheat but lignification after infection is considered important (Ride and Barber, 1987). In this study, FTIR on bulk samples and cross sections of rachis effectively differentiated the cell wall composition between SU3 and MM; the most indicative peaks between cultivars are related to lignin, cellulose, and hemicellulose. These results are consistent with our previous observations (Lahlali et al., 2015), and together showed that FTIR spectroscopy may be used to help identify FHB resistant germplasms based on a range of biochemical differences in fingerprint regions (Alonso-Simon et al., 2004; Lahlali et al., 2015). FTIR has been used to identify biochemical modifications in plants under environmental and biotic stresses (Alonso-Simon et al., 2004; Erukhimovitch et al., 2010; Lahlali et al., 2015). In this study, synchrotron-based FTIR analysis underlines increased amount of lignin, pectin, and hemicellulose in the cell wall of epidermis and highlights these structural components may play a role in the cell wall reinforcement. Interestingly, on the rachis cross sections, the focal plane array imaging also identified a new band around 1710 cm^−1^ related to oxidative stress as a response following the invasion of epidermis and vascular bundles. Because this band was much more intense in MM than in SU3, it may serve as another biomarker for FHB resistance.

### Bulk XRF spectroscopy for nutrients composition in rachis

Although the disease resistance in plants is primarily a function of genetics, the ability of a plant to express its genetic potential can be affected by nutrition and nutrients can be important factors in disease resistance (Agrios, 2005). Interactions between plants, nutrients, and pathogens can be complex and are not well-understood. Essential nutrients have a major role for plants to develop strong cell walls which can affect disease severity (Huber and Graham, 1999; Steinkellner et al., 2005), and the differences in nutrients composition can possibly influence the type II resistance of rachis to FHB. The X-ray analysis has many potential applications in studying plant-pathogen interaction, but the use has rarely been reported due likely to limited access to the equipment. The current data showed that K, Zn, and Fe are present in greater quantities in the rachis of both wheat cultivars. The function of metal ions in plant disease resistance varies; it is known that the fungal-spore germination and plant infection is stimulated by compounds exuded from the plant (Brenner and Romeo, 1986; Steinkellner et al., 2005). In this study, the Ca content was substantially higher in SU3 than in MM after infection, which may indicate the relevance of this element to FHB resistance. As known, Ca play an essential role in formation of healthy and stable cell walls (Dordas, 2008; War et al., 2012). The divalent Ca also plays critical role in signaling transduction upon infection through the calcium-dependent protein kinases pathway (Romeis et al., 2001) and inhibits the formation of cell wall degrading enzymes. In a recent study, an increase in cellular Ca concentration was found to be one of the earliest events of induced plant defense response against many pathogens (Thuleau et al., 2013). The authors highlighted that sphingoid long-chain bases and Ca ions may be inter connected to regulate cellular processes leading to plant susceptibility or resistance mechanisms. The current results also suggest that K, Fe, Zn, Si, and Mn possibly play a role in the resistance to FHB. The critical role of K in plant stress response has been recognized (Wang et al., 2013). Potassium is an essential element affecting most of the biochemical and physiological processes relating to plant growth and metabolism (Dordas, 2008; Wang et al., 2013), altering the host-parasite compatibility by changing the interactive environment. As a facultative parasite, the FHB pathogen invades senescing tissues more rapidly and may create the condition by releasing toxins. Nutrients which support the metabolic activities of host cell and delay the senescence of tissue would potentially increase the resistance or tolerance of plant to facultative parasites by promoting the development of thicker outer walls in epidermal cells (Agrios, 2005). The balance between K and other elements is also important for resistance. For example, the ratios of N/K and K/Ca may affect the susceptibility of plant to diseases (Marschner, 1995). A substantial increase of other elements, including Fe, Zn and Mn in inoculated relative to control MM, may also suggest their involvement in the susceptibility to FHB. Fe is essential for the growth of almost all living organisms; it acts as a catalyst in many metabolic processes such as respiration and photosynthesis (Kieu et al., 2012). Fe can also activate the enzymes involved in plant infection by pathogen or host defense response by promoting antimycosis. Fe does not seem to affect the lignin synthesis, however Fe is a component in peroxidase which stimulates enzymes involved in lignin biosynthesis pathways (Graham and Webb, 1991). Fe appears to be required for three critical defense responses: the formation of cell wall appositions, oxidative burst, and production of pathogenesis-related proteins (Huber and Jones, 2013). In this study, the amount of Fe is very high in SU3 but increased also in infected MM rachises, which may indicate its involvement in infection but its role in FHB resistance is uncertain based on these results. The micronutrient Mn has been linked to the resistance to foliar diseases (Dordas, 2008) while the contribution of Zn to disease resistance is uncertain, but positive and negative effects suggested, depending on the disease (Graham and Webb, 1991; Grewal et al., 1996). Therefore, the higher Mn content in MM relative to that in SU3 and its further increase in MM after inoculation may point to its relatedness to susceptibility of FHB. This observation is in contrast to some of the reports which suggested the importance of Mn in resistance to other diseases (Graham and Webb, 1991; Huber, 1996; Hammerschmidt and Nicholson, 2000; Vidhyasekaran, 2004). Despite its uncertain role in plant disease resistance, Zn plays an important role in proteins and starch biosynthesis. At low concentrations, Zn induces amino acids but reduces sugars in plant tissues (Dordas, 2008). It is also involved in membrane protection against oxidative damage through the detoxification of superoxide radicals (Cakmak, 2000). However, the increase in Zn content during infection of MM links this element more to susceptibility than resistance to FHB. Surprisingly, silicon presence in infected SU3 at 10 DPI, may indicate its crucial role in the resistance to FHB. Although, different reports highlight the importance of Si in plant disease resistance against pathogenic fungi, but the mechanism of Si in plant resistance to diseases is still unclear (Fauteux et al., 2005, 2006).

Comparative FTIR and XRF spectroscopy analyses indicate that lignin, cell wall polymers such hemicellulose as well as nutrients composition in rachis like K, Ca, and Fe are possibly important factors contributing to the strength of cell wall and consequently the type II resistance to FHB. Further investigations on the localization of these nutrients on phloem, xylem, and companion cell walls may better identify their roles in FHB resistance. The results from this work demonstrate the capability of synchrotron-based technique to assess plant histological changes relating to disease resistance mechanisms. Some of the recent studies used XRF to investigate foliar nutrient changes during infection (Pereira and Milori, 2010; Tian et al., 2014) or caused by fungal endophytes (Gonzalez-Chavez et al., 2014; Nayuki et al., 2014). Considering the importance of nutrient composition in several plant biochemical and physiological processes (Dordas, 2008) and based on our findings on wheat rachis, we believe that these synchrotron-based techniques can revolutionize the field of plant-microbe interactions by identifying nutrients involved in plant response mechanisms to abiotic and biotic stresses based on their localization, distribution, and motility at cellular spatial scale.

## Conclusion

This work is the first report using chemometric tools to determine the differences in biochemical and nutrients composition in the tissue of resistant and susceptible hosts. The changes identified in chemical composition following the infection with FHB during this study may be used as biomarkers for screening FHB resistance. The FTIR spectroscopy and imaging indicated the role of cell wall lignification in FHB resistance associated with SU3, and changes in cell wall composition and cell integrity were substantially more pronounced in epidermis and vascular bundles of the susceptible MM after infection. The oxidation stress band (~1710 cm^−1^) was more intense in MM than in SU3 after inoculation. XRF identified higher increase in the elements Ca, K, Si, and Fe in SU3 than in MM after infection, indicating possible involvement of these nutrients in FHB resistance. Further investigation is required to better characterize the roles of these nutrients in FHB resistance based on their localization, distribution, and motility at cellular levels, and use them as biochemical markers for screening FHB resistance.

## Author contributions

RL, LW, PF, and CK conceived this research. RL, CK, and SK collected and analyzed the data. NS, MK participated in sample preparation (microtome sectioning) and in fluorescence microscopy. DM helped during XRF data collection. LF helped in plant growth and inoculation. GS and JL helped in collecting confocal fluorescence data. CK, GP, and PF supervised the work. RL wrote the manuscript and all authors contributed to the manuscript revision.

### Conflict of interest statement

The authors declare that the research was conducted in the absence of any commercial or financial relationships that could be construed as a potential conflict of interest.

## Supplementary material

Figure S1 **Asymptomatic and symptomatic infected rachis of the resistant and susceptible cultivars Sumai3 and Muchmore in comparison with control rachis at 10 days post-inoculation with FHB**. Scale bar 4 mm. **(A)**: control Sumai3, **(B)**: inoculated Sumai3 with FHB, **(C)** control Muchmore, and **(D)** inoculated Muchmore with FHB.

Figure S2 **Fluorescent (A–D) microscope images from cross section (10 μm) of control and inoculated wheat cultivars Sumai3 (SU3, A,B) and Muchmore (MM, C,D) after 4 days of infection with FHB**. Red arrows show changes in cell wall thickness following the pathogenic infection with FHB. Scale bar = 75 μm.

Figure S3 **Confocal microscope movie showing cell wall structures of the infected wheat rachis of the resistant cultivar Sumai3 (A) and the susceptible cultivar Muchmore (B) with Fusarium head blight at 4 days post-inoculation**.
