# Supplementary figures and images for: Cell Wall Biomolecular Composition Plays a Potential Role in the Host Type II Resistance to Fusarium Head Blight in Wheat
Source: Front Microbiol. 2016 Jun 27;7:910. doi: 10.3389/fmicb.2016.00910 (PMC4921494; doi:10.3389/fmicb.2016.00910)

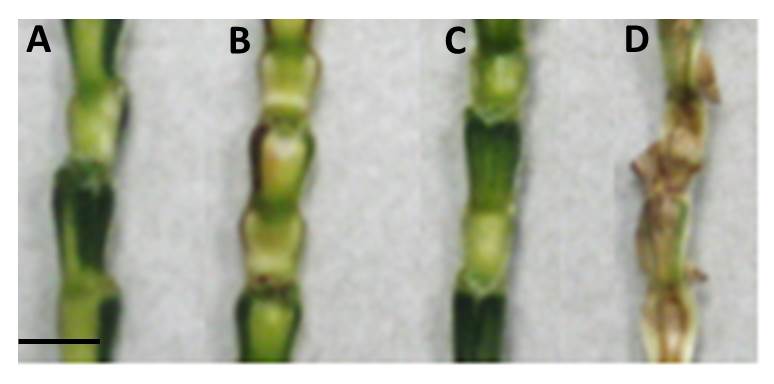

Supplement: Figure S1 — Asymptomatic and symptomatic infected rachis of the resistant and susceptible cultivars Sumai3 and Muchmore in comparison with control rachis at 10 days post-inoculation with FHB. Scale bar 4 mm. (A): control Sumai3, (B): inoculated Sumai3 with FHB, (C) control Muchmore, and (D) inoculated Muchmore with FHB. [file Image1.jpg]

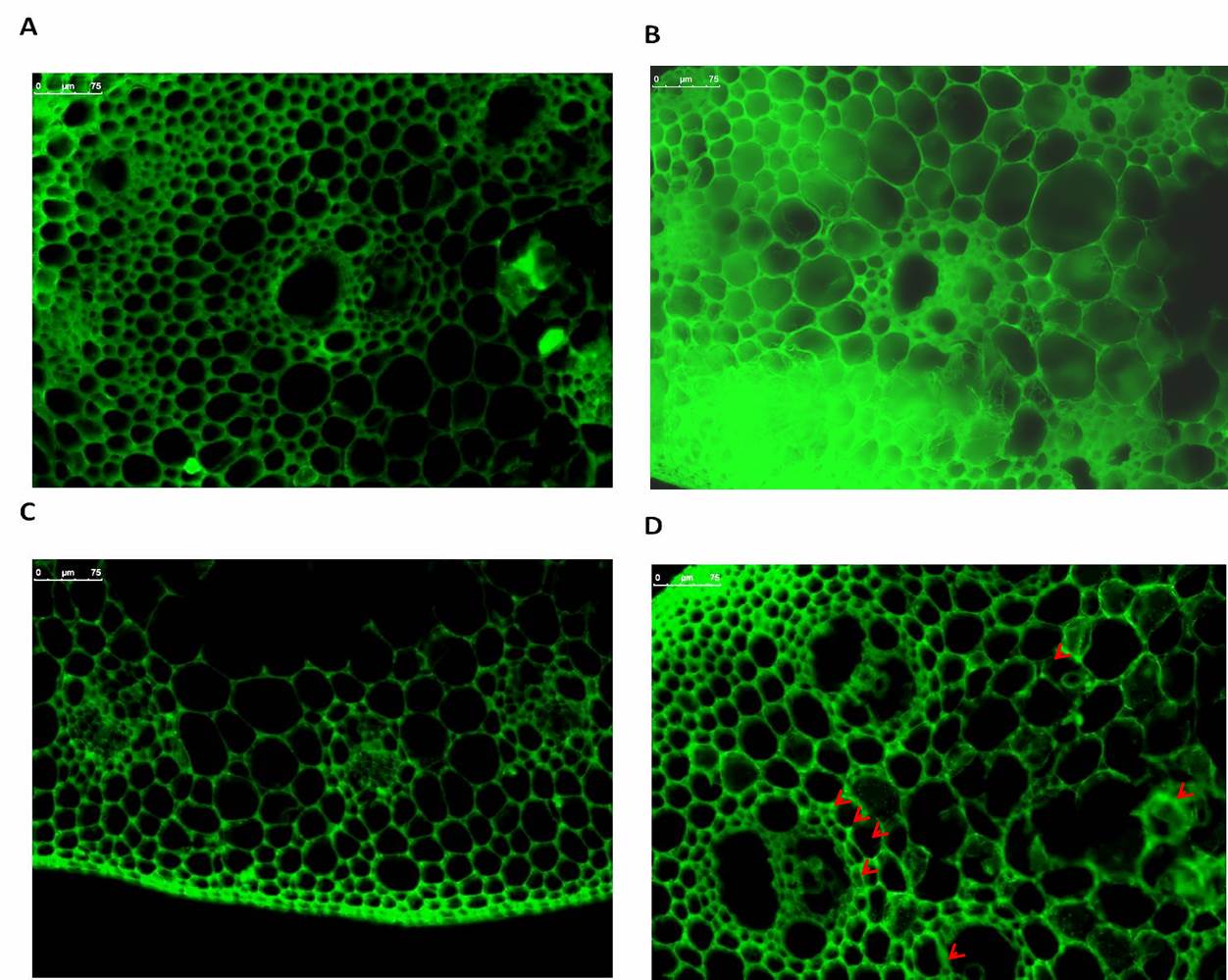

Supplement: Figure S2 — Fluorescent (A–D) microscope images from cross section (10 μm) of control and inoculated wheat cultivars Sumai3 (SU3, A,B) and Muchmore (MM, C,D) after 4 days of infection with FHB. Red arrows show changes in cell wall thickness following the pathogenic infection with FHB. Scale bar = 75 μm. [file Image2.jpeg]
