# Supplementary material for: Cell Wall Biomolecular Composition Plays a Potential Role in the Host Type II Resistance to Fusarium Head Blight in Wheat
Source: Front Microbiol. 2016 Jun 27;7:910. doi: 10.3389/fmicb.2016.00910 (PMC4921494; doi:10.3389/fmicb.2016.00910)
Supplement: Figure S3 — Confocal microscope movie showing cell wall structures of the infected wheat rachis of the resistant cultivar Sumai3 (A) and the susceptible cultivar Muchmore (B) with Fusarium head blight at 4 days post-inoculation. [file Image3.pdf]

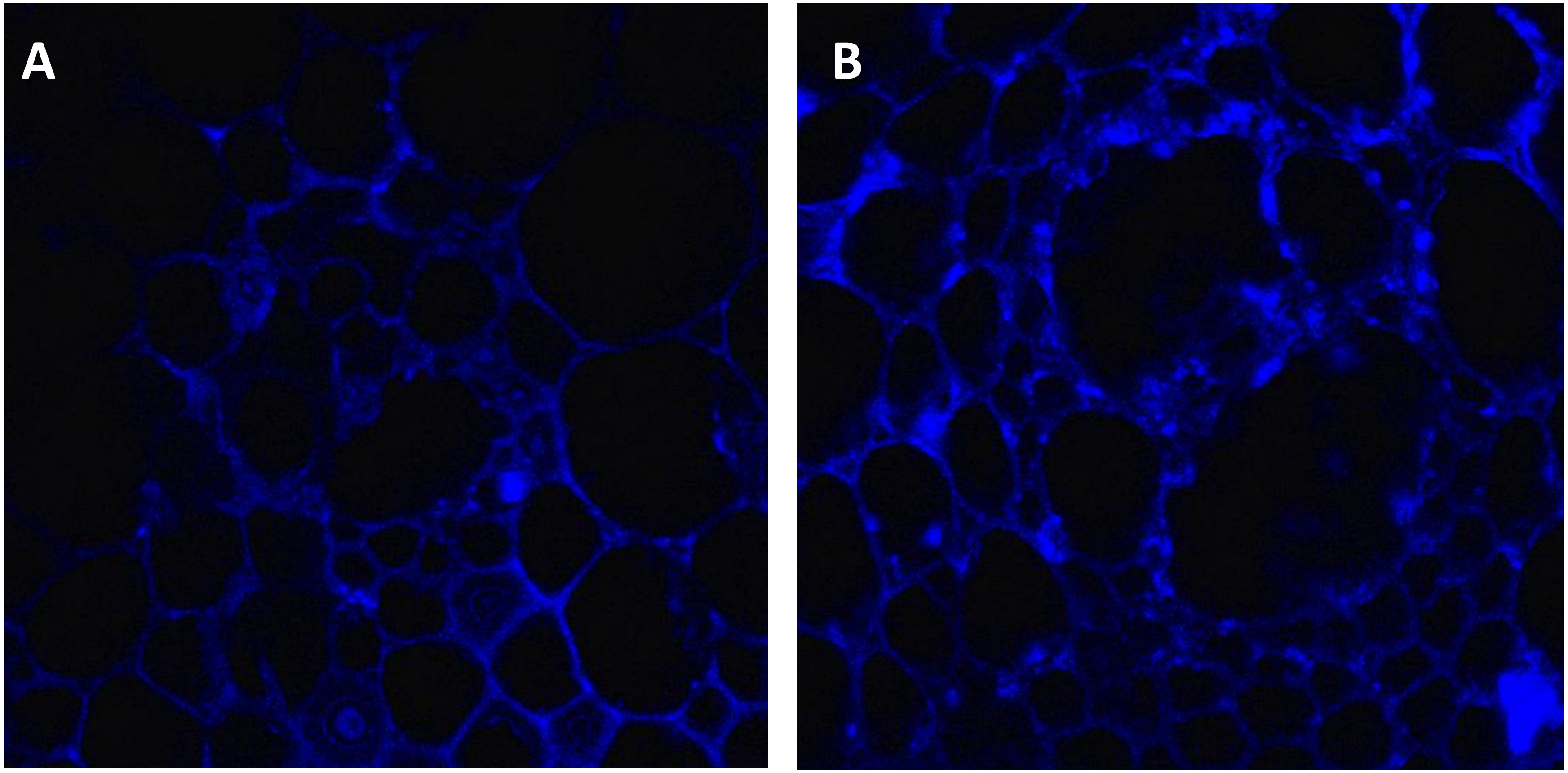

Figure S3: Confocal microscope movie showing cell wall structures of the infected wheat rachis of the resistant cultivar Sumai3 (SU3, A) and the susceptible cultivar Muchmore (MM, B) with Fusarium head blight (FHB) at 4 days post-inoculation period.
